# Tele-Rehabilitation for Postural Control by Means of Virtual Reality Rehabilitation System in an Adolescent With Motor Disorder: A Case Study

**DOI:** 10.3389/fpsyg.2021.720677

**Published:** 2021-10-14

**Authors:** Valentina Menici, Veronica Barzacchi, Silvia Filogna, Elena Beani, Francesca Tinelli, Giovanni Cioni, Giuseppina Sgandurra

**Affiliations:** ^1^Department of Developmental Neuroscience, Istituto di Ricovero e Cura a Carattere Scientifico (IRCCS) Fondazione Stella Maris, Pisa, Italy; ^2^Department of Clinical and Experimental Medicine, University of Pisa, Pisa, Italy

**Keywords:** tele-rehabilitation, motor disorder, postural control, balance, medical device, Virtual Reality Rehabilitation System, COVID-19

## Abstract

The coming of an unforeseen and hostile event such as the COVID-19 pandemic has brought about various changes in everyone's daily life. During the lockdown period, a huge number of restrictions were imposed, hence interrupting a wide range of activities previously proven to be necessary for some people. Due to the circumstances, rehabilitation treatments for children with neurodevelopmental disorders have been suspended, resulting in consequent distress for the children themselves and their parents. To overcome this problem, a tele-rehabilitation approach has proven to be an excellent solution to give continuity to children's rehabilitation. The tele-rehabilitation approach allows access to rehabilitation services directly from home. During lockdown due to the COVID-19 pandemic, the Virtual Reality Rehabilitation System (VRRS) HomeKit, developed by Khymeia, was employed as a rehabilitation system for the treatment of posture and balance of an adolescent with disabilities for the first time ever. Specifically, a 17-year-old female patient was enrolled and evaluated by the clinical staff before and after the home-training sessions. The system was delivered to the patient's home so that she could perform the tasks in a familiar environment, while under the supervision of the clinical staff. Through a specific platform, using different modules of the system, therapists could remotely check that the proposed exercises were properly performed and provide feedback and/or increase the difficulty according to the patient's needs and progress. Therefore, the treatment performed was carried out at home in a personalized, intensive, and playful way; characteristics do not present in a traditional treatment. Our results are promising and demonstrate both the efficacy of rehabilitation exercises carried out at home and the feasibility of home-based rehabilitation, when using the VRRS HomeKit even with adolescents. The VRRS HomeKit presents some limitations, such as the need to have the line connection and free space at home, the presence of technical issues, and the education of parents and patients to understand the instructions. Despite the limitations, this study provides the basis for continuing the experience of tele-rehabilitation on patients with a motor disorder also by customizing the exercises to their characteristics.

## Introduction

In the last two years, an unexpected Coronavirus (COVID-19) pandemic has spread rapidly around the world driving governments to introduce security measures and restrictions that have led to a period of total closure, known as *lockdown*. This period changed everyone's routine life and, above all, it has had a strong impact on clinical activities and rehabilitation services (Barello and Graffigna, 2020). In this context, those who suffered most were fragile subjects, as children with neurodevelopmental disabilities and their parents, as their rehabilitation care was interrupted (Provenzi et al., 2020).

In this stressful situation, tele-rehabilitation methods have proven to be a good solution; they have provided continuity of children's care, lightened the burden on parents and avoided the spread of contagion. The tele-rehabilitation approach is not an innovation that came into being in this pandemic, but it has always been a useful method to gain access to rehabilitation services by overcoming not only those difficulties due to the geographical distance, but also motor impairment in patients with neurological and neurodegenerative diseases. However, despite being recognized as a promising solution, tele-rehabilitation presents some difficulties regarding it being incorporated into clinical practice (i.e., low acceptability by clinical staff, management costs, etc.) (Touré et al., 2012). However, the pandemic has forced hospitals to increase these services and therapists have done everything possible to immediately recommence serving all those families previously supported (Camden and Silva, 2021).

In most cases, the technologies available for motor and cognitive tele-rehabilitation are employed in adults affected by stroke (Lohse et al., 2014; Maresca et al., 2020). One of these systems was provided by Khymeia (Padova, Italy), namely Virtual Reality Rehabilitation System (VRRS) HomeKit, through which patients underwent modifiable rehabilitative exercises according to their needs. Likewise, Camden et al. (2020) and Caprì et al. (2021) summarized tele-rehabilitation interventions in children and young adults with disabilities but these applications are still few. In this context, Sgandurra and co-workers developed two platforms (called CareToy and Tele-UPCAT) dedicated to intensive home rehabilitation programs (Sgandurra et al., 2017, 2018a,b) to improve the motor outcome of infants, children, and young adults, respectively. Following these experiences, the same research group started to use the VRRS HomeKit for the tele-rehabilitation of children, as part of an Italian Ministry of Health project.

Based on this, the general objective of our study was to evaluate the feasibility of tele-rehabilitation with the VRRS HomeKit for the personalized, intensive, and playful treatment of static posture and balance capabilities of an adolescent, at home, during the COVID-19 pandemic. In particular, the aims were threefold: (i) to provide a customized goal-directed home training focused on the improvement of balance abilities by means of the VRRS system; (ii) to identify and apply standardized clinical scales that evaluate the patient's gross motor skills, posture maintenance, and functional and postural balance before and after the home treatment; and (iii) to monitor the trend of the training by analyzing the data of two specific exercises performed periodically during the entire training period.

## Materials and Methods

### Patient Profile

In this explorative study, a 17-year-old female patient (160.00 cm tall and weighed 72.00 kg) of the IRCCS Fondazione Stella Maris was recruited. She has a diagnosis of myopathy, to date genetically and histopathologically undefined, which affects the proximal girdles especially. She has a moderate intellectual disability, even if her adaptive functioning was in the mild level. The clinical picture is characterized by dysmetria of the right lower limb and attitude of pelvis rotation toward the left, with sacral hemilumbarization of S1. Her motor skills are characterized by an impaired gait pattern and difficulties in balance, fatigue, motor awkwardness, and a tendency to stumble. She wears eyewear to correct the vision. Other features of her disorder include a slight fluctuation of emotional states. The patient had no other relevant comorbidities.

After the functional observation, she was instructed to undergo rehabilitation therapy for postural control and static and dynamic balance improvement. She was classified as having the second level of the International Standard Classification of Education and her experience with computers and technologies was reported as averagely high. Her clinical traditional rehabilitative therapy, provided by the National Health System (NHS), consisted of one session of physiotherapy a week, which had been interrupted two months before the VRRS training began.

### Intervention Protocol

The clinical staff evaluated the girl in the hospital with standardized outcome measures (described below) before starting the training (T0, baseline), immediately after the training (T1, in the week after the end of the training), and two months after the end of the training (T2).

**Figure 1:**
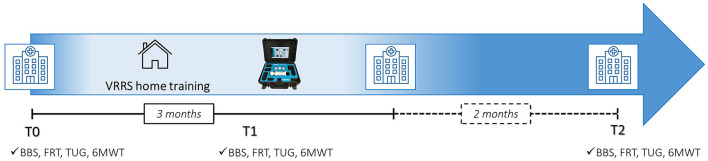
Timeline of the study. BBS, berg balance scale; FR, functional reach test; TUG, timed up and go; 6MWT, six-minute walk test; T0, baseline; T1, in the week after the end of the training; T2, 2 months after the end of the training.

After the T0 assessment, the VRRS system was delivered to her home. The training was planned for three months, with daily activities lasting 40 to 50 min, organized in different exercises (with different goals); the girl was asked to perform the training for a minimum of three days per week (Figure 1).

### Procedures

The VRRS environment is composed of several system units and external modules which allow the administration of rehabilitation protocols, both in presence (VRRS central hub) and at a distance (home tablet). In the current study, the VRRS HomeKit was used and connected with its stabilometric balance to evaluate the patient's static and dynamic equilibrium, that acquired, measured, and evaluated the static postural data by using integrated load cells. In particular, the patient performed the exercises standing on the balance board, which detected the displacements of the Centre of Pressure (COP) with respect to the two medial-lateral and antero-posterior axes. The system generated virtual images which changed position on the screen according to the weight shift. Finally, an integrated high-quality webcam enabled therapists to observe the patient's movement during the training.

The connection procedure for the remote control and for storing the data in an online platform was based on an Information and Communication Technology (ICT), which merely required access to the Internet. Data were also saved on the device itself.

### Assessment Tools

The outcome measures selected in this study were:

- The Berg Balance Scale (BBS): measures balance with different tasks in people with varying conditions and disabilities. It is a reliable and valid scale with high intra- and inter-rater relative reliability (Berg et al., 1992; Downs et al., 2013; Godi et al., 2013; Alghadir et al., 2018).
- The Functional Reach (FR) test: a functional assessment of dynamic balance evaluated in the standing position and measures the maximum distance an individual can reach with an anterior shift with a fixed base of support (Duncan et al., 1990).
- The Timed Up and Go (TUG): functional dynamic balance test which measures the time it takes for the patient to stand up from a chair, walk 3 m, turn around, walk back, and sit down (Verbecque et al., 2019).
- The Six-Minute Walk Test (6MWT): evaluates how far the subject walks along a 30-m corridor for 6 min (Cacau et al., 2016).

### Training Session

The VRRS HomeKit was delivered to the family's house and clinical and technical assistance were provided through a printed manual and video calls. The girl and her parents were initially trained on how to use the system by the therapists with daily video call sessions; subsequently, the calls were scheduled for two or three times a week. The training was followed by therapists through an *ad-hoc* platform, enabling them to monitor performance, update the training, and download and analyze data. The online sessions with the therapist in video calls were performed twice a week and other sessions were executed independently offline by the girl.

**Figure 2:**
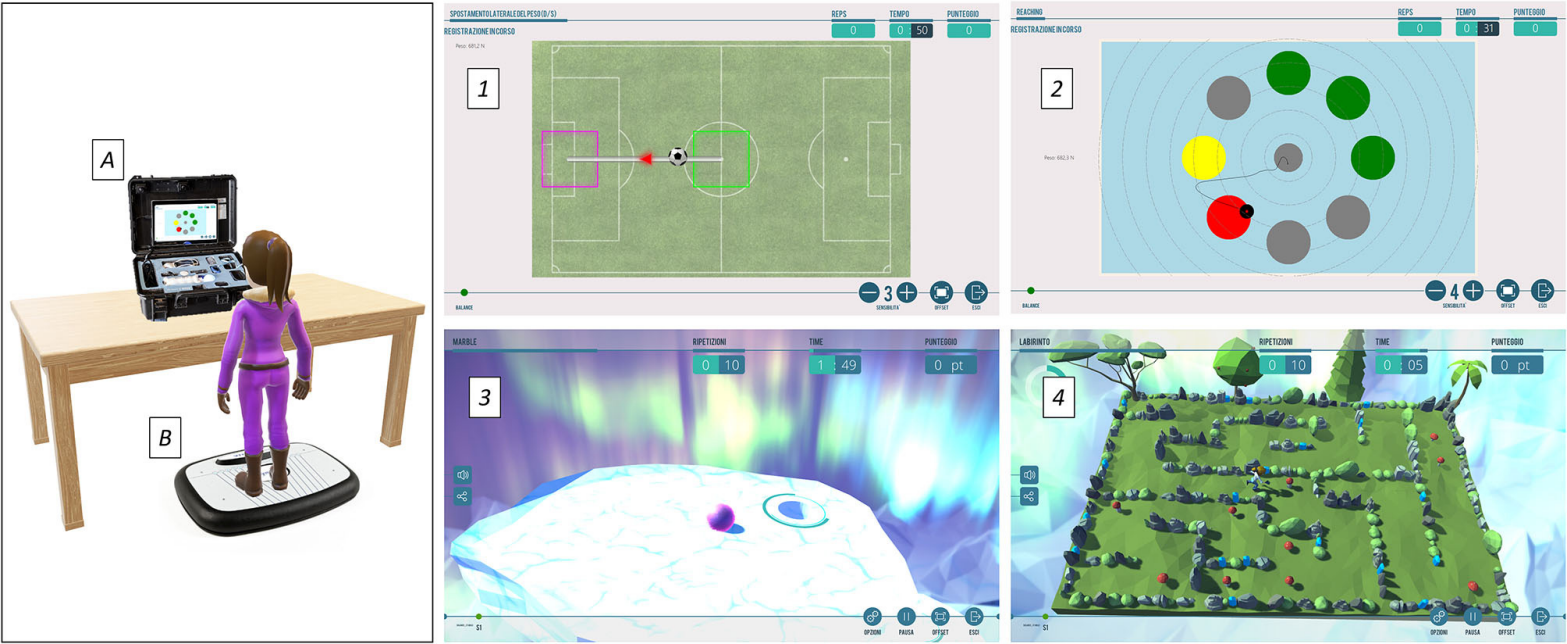
On the **(left)**, the setting of the home training with the VRRS HomeKit (A) and the stabilometric balance (B). On the **(right)**, some examples of the proposed exercises: (1) Reaching points (R), (2) Reaching and (3), (4) two exercises of 3D environment. The figures of exercises are shown by permission of Khymeia.

The therapists planned the first period of training based on the T0 evaluation, tailoring the training according to the patient's functional skills and the goals to be achieved. The training exercises (from 6 to 10 exercises per day, lasting from 2 to 8 min) included postural tasks, starting from unidirectional load shifts up to moving in all directions and in three-dimensional (3D) environments (Figure 2), thus increasing the difficulty level. Among the range of rehabilitation exercises available, the therapists selected exercises that remained unchanged throughout the entire training period (i.e., “Reaching points [Right, R]” exercise where the patient had to move her COP to reach one target along a visible path on the screen), while others (i.e., “Reaching” exercise where the patient had to reach an increased number of targets, placed around her COP in different directions, trying to take the most direct path possible) were modified periodically according to the patient's needs and progress, either by increasing the difficulty (e.g., increasing the number of targets to be reached or the stabilometric balance sensitivity) or activating feedbacks (auditory, visual, etc.).

### Data Collection and Statistical Analysis

All clinical data of the subject were collected, and all clinical assessments were video-recorded and blindly scored by a therapist, subsequently.

The data obtained from the T0, T1, and T2 clinical evaluations were calculated by evaluating the difference (delta values). These differences were compared with the data in the literature on individual standardized tests regarding the Minimal Detectable Change (MDC), that is the minimum difference that reflects the real change of a patient between the time points of two assessments.

For the training data, the VRRS parameters analyzed were for Reach points (R) exercise the Mean Time in ms (MT), Time Spent in s (TS) and Average Score in percentile (AS) while for the Reaching exercise the Mean Time in ms (MT), the Average Ratio between the distance covered and the minimum distance to be covered (AT), and the Number of Targets Achieved (NTA).

Statistical analysis was carried out with the Statistical Package for Social Sciences software (SPSS, version 20.0, IBM^®^, Italy). The level of statistical significance was set at *p* < 0.05. Spearman's Rho index was calculated between the VRRS training data and the sessions.

## Results

### Training Results

The training was carried out over 3 months, by the end of which the subject had executed 48 sessions for a total of 23 h and 48 min with a mean daily session of 30.23 ± 0.01 min. The total number of executed exercises was 387 with a mean daily of 8.06 ± 1.78.

### Assessment Tools

Table 1 shows the results of the clinical assessments performed at all the evaluation times planned in the study. The MDC for each measurement is reported in the last column.

**Table 1:** Results of assessment tools.

**Assessment tools**	**T0**	**T1**	**T2**	**ΔT**	**ΔT**	**ΔT**	**MDC**
				**(T1–T0)**	**(T2–T1)**	**(T2–T0)**	
BBS	52	56	56	4	0	4	2.7
FR	20 cm	27 cm	26 cm	7 cm	**–**1 cm	6 cm	2.3 cm
TUG	6.80 s	5.67 s	5.87 s	**–** 1.13 s	0.2 s	**–**0.93 s	3.5 s
6MWT	445.70 m	515.00 m	518.60 m	69.30 m	3.6 m	72.90 m	50 m

*BBS, berg balance scale; FR, functional reach test; TUG, timed up and go; 6MWT, six-minute walk test; T0, baseline; T1, in the week after the end of the training; T2, 2 months after the end of the training; MDC, minimal detectable change*.

### Training Exercises Results

**Figure 3:**
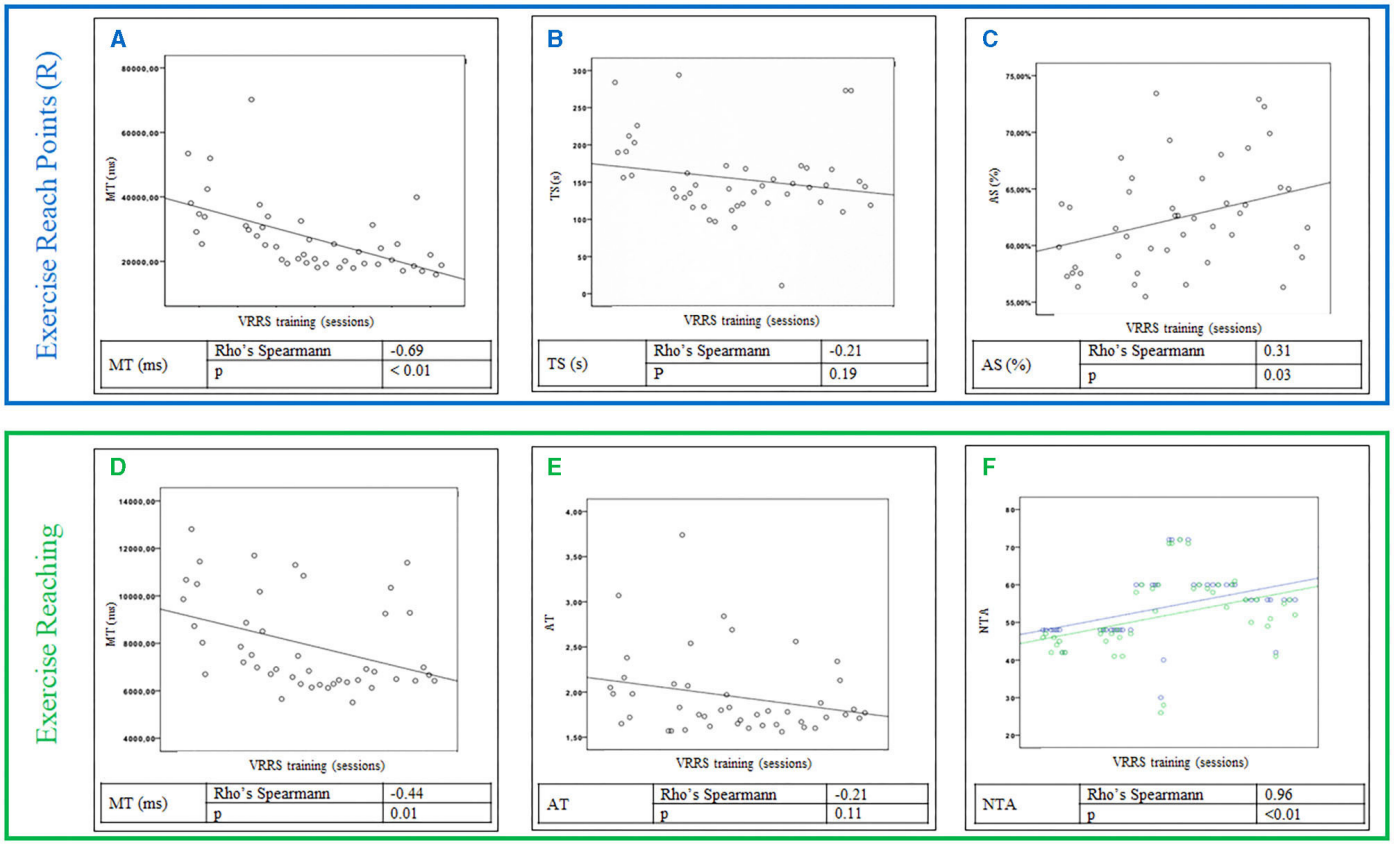
Upper section, Reach points (R) exercise results: values of the Mean Time (MT) to reach the target **(A)**, the Time Spent (TS) **(B)** and the Average Score (AS) **(C)** obtained in relation to the session performed during the VRRS training. Lower section, Reaching exercise: Values of the Mean Time (MT) to reach the target **(D)**, the Average Ratio (AT) between the distance covered and the minimum distance to be covered **(E)**, both in relation to the session performed, and the Number of Targets Achieved (NTA) (in green) and those to be achieved (blue) **(F)** during the VRRS training.

The Reach points (R) exercise was performed 46 times during the training period. Figure 3A shows the values of the MT spent to reach the target in relation to the sessions performed; the data are statistically significant, and an inversely proportional change over time can be observed. Figure 3B presents the TS in relation to the sessions performed, which are not statistically significant. Figure 3C represents the AS obtained in relation to the sessions conducted. This value is statistically significant as a directly proportional variation can be observed.

The Reaching exercise was performed 46 times during the entire training period, and it was the most modified exercise. The modifications made by the therapists only concern the number of targets to be achieved. Figure 3D presents the values of the MT spent to reach the target in relation to the sessions carried out. They are statistically significant as an inversely proportional variation is noted. Figure 3E describes the data of the AT in relation to the sessions performed; the data are not significant. Finally, Figure 3F shows the correlation between NTA and those to be achieved, which are statistically significant, and the correlation is directly proportional.

## Discussion

Since the use of ICT in medicine has increased, researchers are interested in how technology can be useful to advance access to rehabilitation services for individuals with disabilities (Edirippulige et al., 2016; Peretti et al., 2017; Camden et al., 2020).

Although several systems allow tele-rehabilitation practices, most of them have been designed for adults, as in the case of VRRS systems. The latter have already been employed for children's rehabilitation inside hospitals (Olivieri et al., 2013), but to our knowledge, this is the first time where the VRRS HomeKit was applied for adolescent home rehabilitation in the field of postural control.

The COVID-19 pandemic has offered an opportunity to implement the use of tele-rehabilitation during the lockdown. Our adolescent patient was able to avail of personalized, intensive, innovative, and playful customized rehabilitation training although she lived far from the hospital. The VRRS environment permitted us to remotely plan and monitor her training, in an online/offline mode, by carefully selecting and customizing the exercises to promote balance and postural control.

The patient showed excellent adherence to the treatment performing many sessions, and she completed all the exercises provided in the daily sessions. Her compliance was high; during the training, the number of exercises progressively increased over time from 4 to 10. Cognitive impairment did not interfere with her ability to perform the training; she was able to understand the instructions and managed the system autonomously. Furthermore, she reported that the system was easy to use, she felt free and confident using it, performing the planned exercises with pleasure and enjoyment; indeed, she would have liked to continue with this treatment.

The adolescent was evaluated before and after the training with some standardized scales to measure gross motor skills, posture, maintenance, and functional and postural balance. In the literature, some assessment tools have been reported which evaluate both static and dynamic balance, especially for older patients but they can be adapted to subjects with congenital motor disorders (Niznik et al., 1996; Steffen et al., 2002; Downs et al., 2013; Carey et al., 2016; Alghadir et al., 2018). We, therefore, selected BBS, FR, TUG, and 6MWT and administered them at all time points in this study. Results indicate that three months of training improved postural and balance abilities, with points higher than the MDC; namely in the tests of balance, speed, anterior-posterior and medial-lateral weight shift, and postural control.

We analyzed the intra training data of two specific exercises executed periodically during the entire training period. In the “Reaching points (R)” exercise, the MT to reach the target was significantly reduced thanks to a better weight balance on the lower limbs, resulting in a faster movement. Conversely, the result of the TS parameter was not statistically significant. Since it measures the time from the start of the exercise to the achievement of the last goal, it does not consider possible interruptions, distractions, or Internet connection problems. The result is therefore not reliable because of possible bias occurring during the execution of the task. The third parameter analyzed, the AS, is statistically significant and demonstrates that the patient was able to directly reach the targets without balancing errors, hence obtaining a higher score. The significant results of both MT and AS are correlated because the execution of correct and controlled movements allowed the patient to reach the targets faster.

The second exercise, “Reaching,” was modified by increasing the difficulty during the training. The MT parameter, which was independent of the total number of targets to be achieved, was also considered for this exercise. These results were statistically significant. In fact, during the training, the patient showed better ability in the load transfers and weight balancing in various directions. The NTA parameter during the training was very significant and the patient was able to reach the goals over time, despite the increase in target number.

Therefore, the data obtained show significant performance improvements both in the execution of the exercises during training and in clinical evaluations.

## Conclusion

This study is promising and demonstrates both the efficacy of rehabilitation exercises carried out at home and the feasibility of home-based rehabilitation, using the VRRS HomeKit.

In the present study, we found some limitations that need to be highlighted and overcome in the future for continuing the experience of tele-rehabilitation and improving it.

First, at least an ADSL line connection is essential to carry out the training, so that therapists can make video calls with the patient and clearly see the exercise graphics, especially those in 3D. Moreover, when some technical issues occurred, the therapists required technical support from the engineers. Additionally, to use this system, it was necessary to reorganize the space in the house as the system and the balance had to be placed in an easily accessible area so that the girl could stand and move freely. A further limitation of the study is that the long-term effects were not investigated, but we only considered the follow-up at T2; it might be useful to evaluate these in future works.

From these very preliminary data, it is not possible to perform a health economic analysis, even if studies available in the literature (Wong et al., 2021) confirm that virtual care could reduce health care costs. As reported in the literature, sociodemographic characteristics and some health conditions can influence the ability to use technology (Scott Kruse et al., 2018; Crawford and Serhal, 2020; Nouri et al., 2020), indeed, not all patients have easy access (Wong et al., 2021), although it was not the case in this study as reported above. The option of having a patient's family member, educated in using it, to facilitate the virtual session is useful (Appireddy et al., 2019).

Finally, the patient reported many comments, and it would be interesting to create an *ad-hoc* questionnaire to evaluate the feasibility of this treatment.

Despite the aforementioned limitations, this study shows how the system seems to be highly flexible thanks to the customization of the exercises, according to the patient's skills. Moreover, having the system at home allowed the family to better organize their time both to carry out the training and to increase the frequency and intensity of the rehabilitation. The promising results, combined with these aspects, show the treatment feasibility and effectiveness.

To date, the VRRS environment represents one of the several available home-based systems, the potential and the benefits of which had already been implemented before the pandemic (Luis Pérez Medina et al., 2019).

In conclusion, this study highlights the opportunity afforded by tele-rehabilitation under normal conditions but also in unpredictable situations, such as the COVID-19 pandemic.

## Data Availability Statement

The raw data supporting the conclusions of this article will be made available by the authors, without undue reservation.

## Ethics Statement

The studies involving human participants were reviewed and approved by Tuscany Pediatric Ethics Committee. Written informed consent to participate in this study was provided by the participants' legal guardian/next of kin.

## Author Contributions

GS and GC conceived the idea for this original research. FT carried out the enrollment of the participant. VM did the assessment with the standardized outcome measures. VM and VB led the training sessions. GS analyzed data. VM, VB, SF, and EB conceived and prepared the manuscript. All the authors read, critically revised, and approved the final manuscript.

## Funding

The Italian Ministry of Health partially funded the present work by means of a project entitled Development and Implementation of an Italian Network of Tele-Neuro-Rehabilitation for Continuity of Care. It was also partially funded by Grant Rete RIN 2019 and 5 X 1000 Health Research 2017.

## Conflict of Interest

The authors declare that the research was conducted in the absence of any commercial or financial relationships that could be construed as a potential conflict of interest.

## Publisher's Note

All claims expressed in this article are solely those of the authors and do not necessarily represent those of their affiliated organizations, or those of the publisher, the editors and the reviewers. Any product that may be evaluated in this article, or claim that may be made by its manufacturer, is not guaranteed or endorsed by the publisher.

## References

[B1] Alghadir A. H. Al-Eisa E. S. Anwer S. Sarkar B. (2018). Reliability, validity, and responsiveness of three scales for measuring balance in patients with chronic stroke. BMC Neurol. 18:141. 10.1186/s12883-018-1146-9 30213258 PMC6136166

[B2] Appireddy R. Khan S. Leaver C. Martin C. Jin A. Durafourt B. A.. (2019). Home virtual visits for outpatient follow-up stroke care: cross-sectional study. J. Med. Internet Res. 21:e13734. 10.2196/13734 31593536 PMC6803894

[B3] Barello S. Graffigna G. (2020). Caring for health professionals in the COVID-19 pandemic emergency: toward an “epidemic of empathy” in healthcare. Front. Psychol. 11:1431. 10.3389/fpsyg.2020.01431 32581986 PMC7296111

[B4] Berg K. O. Wood-Dauphinee S. L. Williams J. I. Maki B. (1992). Measuring balance in the elderly: validation of an instrument. Can. J. Public Health Rev. 83, S7–S11. 1468055

[B5] Cacau L. de A. P. Santana-Filho V. J. de Maynard L. G. Neto M. G. Fernandes M. Carvalho V. O. (2016). Reference values for the six-minute walk test in healthy children and adolescents: a systematic review. Braz. J. Cardiovasc. Surg. 31, 381–388. 10.5935/1678-9741.20160081 27982347 PMC5144571

[B6] Camden C. Pratte G. Fallon F. Couture M. Berbari J. Tousignant M. (2020). Diversity of practices in telerehabilitation for children with disabilities and effective intervention characteristics: results from a systematic review. Disabil. Rehabil. 42, 3424–3436. 10.1080/09638288.2019.1595750 30978110

[B7] Camden C. Silva M. (2021). Pediatric teleheath: opportunities created by the COVID-19 and suggestions to sustain its use to support families of children with Disabilities. Phys. Occup. Ther. Pediatr. 41, 1–17. 10.1080/01942638.2020.1825032 33023352

[B8] Caprì T. Nucita A. Iannizzotto G. Stasolla F. Romano A. Semino M.. (2021). Telerehabilitation for improving adaptive skills of children and young adults with multiple disabilities: a systematic review. Rev. J. Autism Dev. Disord. 8, 244–252. 10.1007/s40489-020-00214-x

[B9] Carey H. Martin K. Combs-Miller S. Heathcock J. C. (2016). Reliability and responsiveness of the timed up and go test in children with cerebral palsy. Pediatr. Phys. Ther. 28, 401–408. 10.1097/PEP.0000000000000301 27661230

[B10] Crawford A. Serhal E. (2020). Digital health equity and COVID-19: the innovation curve cannot reinforce the social gradient of health. J. Med. Internet Res. 22:e19361. 10.2196/19361 32452816 PMC7268667

[B11] Downs S. Marquez J. Chiarelli P. (2013). The berg balance scale has high intra- and inter-rater reliability but absolute reliability varies across the scale: a systematic review. J. Physiother. 59, 93–99. 10.1016/S1836-9553(13)70161-9 23663794

[B12] Duncan P. W. Weiner D. K. Chandler J. Studenski S. (1990). Functional reach: a new clinical measure of balance. J. Gerontol. 45, M192–M197. 10.1093/geronj/45.6.M192 2229941

[B13] Edirippulige S. Reyno J. Armfield N. R. Bambling M. Lloyd O. McNevin E. (2016). Availability, spatial accessibility, utilisation and the role of telehealth for multi-disciplinary paediatric cerebral palsy services in Queensland. J. Telemed. Telecare 22, 391–396. 10.1177/1357633X15610720 26519377

[B14] Godi M. Franchignoni F. Caligari M. Giordano A. Turcato A. M. Nardone A. (2013). Comparison of reliability, validity, and responsiveness of the mini-BESTest and berg balance scale in patients with balance disorders. Phys. Ther. 93, 158–167. 10.2522/ptj.20120171 23023812

[B15] Lohse K. R. Hilderman C. G. E. Cheung K. L. Tatla S. Van der Loos H. F. M. (2014). Virtual reality therapy for adults post-stroke: a systematic review and meta-analysis exploring virtual environments and commercial games in therapy. PLoS ONE 9:e93318. 10.1371/journal.pone.0093318 24681826 PMC3969329

[B16] Luis Pérez Medina J. Acosta-Vargas P. Rybarczyk Y. (2019). A systematic review of usability and accessibility in tele-rehabilitation systems, in Assistive and Rehabilitation Engineering, ed Rybarczyk Y. (London: IntechOpen), 31–50. 10.5772/intechopen.85869

[B17] Maresca G. Maggio M. G. De Luca R. Manuli A. Tonin P. Pignolo L.. (2020). Tele-neuro-rehabilitation in Italy: state of the art and future perspectives. Front. Neurol. 11:563375. 10.3389/fneur.2020.563375 33101176 PMC7554582

[B18] Niznik T. M. Turner D. Worrell T. W. (1996). Functional reach as a measurement of balance for children with lower extremity spasticity. Phys. Occup. Ther. Pediatr. 15, 1–16. 10.1080/J006v15n03_01

[B19] Nouri S. Khoong E. C. Lyles C. R. Karliner L. (2020). Addressing equity in telemedicine for chronic disease management during the Covid-19 Pandemic. Nejm Catalyst Innov. Care Deliv. 1, 1–13. 10.1056/CAT.20.012332540841

[B20] Olivieri I. Chiappedi M. Meriggi P. Mazzola M. Grandi A. Angelini L. (2013). Rehabilitation of children with hemiparesis: a pilot study on the use of virtual reality. BioMed. Res. Int. 2013, 1–5. 10.1155/2013/695935 24199197 PMC3808096

[B21] Peretti A. Amenta F. Tayebati S. K. Nittari G. Mahdi S. S. (2017). Telerehabilitation: review of the state-of-the-art and areas of application. JMIR Rehabil. Assist. Technol. 4:e7. 10.2196/rehab.7511 28733271 PMC5544892

[B22] Provenzi L. Grumi S. Borgatti R. (2020). Alone with the kids: tele-medicine for children with special healthcare needs during COVID-19 emergency. Front. Psychol. 11:2193. 10.3389/fpsyg.2020.02193 33013567 PMC7509132

[B23] Scott Kruse C. Karem P. Shifflett K. Vegi L. Ravi K. Brooks M. (2018). Evaluating barriers to adopting telemedicine worldwide: a systematic review. J. Telemed. Telecare 24, 4–12. 10.1177/1357633X16674087 29320966 PMC5768250

[B24] Sgandurra G. Beani E. Giampietri M. Rizzi R. Cioni G. (2018a). CareToy-R Consortium Early intervention at home in infants with congenital brain lesion with CareToy revised: a RCT protocol. BMC Pediatr. 18:295. 10.1186/s12887-018-1264-y 30185165 PMC6126039

[B25] Sgandurra G. Cecchi F. Beani E. Mannari I. Maselli M. Falotico F. P.. (2018b). Tele-UPCAT: study protocol of a randomised controlled trial of a home-based Tele-monitored UPper limb children action observation training for participants with unilateral cerebral palsy. BMJ Open 8:e017819. 10.1136/bmjopen-2017-017819 29764869 PMC5961615

[B26] Sgandurra G. Lorentzen J. Inguaggiato E. Bartalena L. Beani E. Cecchi F.. (2017). A randomized clinical trial in preterm infants on the effects of a home-based early intervention with the “CareToy System.” PLoS ONE 12:e0173521. 10.1371/journal.pone.0173521 28328946 PMC5362053

[B27] Steffen T. M. Hacker T. A. Mollinger L. (2002). Age- and gender-related test performance in community-dwelling elderly people: six-minute walk test, berg balance scale, timed up & go test, and gait speeds. Phys. Ther. 82, 128–137. 10.1093/ptj/82.2.128 11856064

[B28] Touré M. Poissant L. Swaine B. R. (2012). Assessment of organizational readiness for *e*-health in a rehabilitation centre. Disabil. Rehabil. 34, 167–173. 10.3109/09638288.2011.591885 21936712

[B29] Verbecque E. Schepens K. Theré J. Schepens B. Klingels K. Hallemans A. (2019). The timed up and go test in children: does protocol choice matter? A systematic review. Pediatr. Phys. Ther. 31, 22–31. 10.1097/PEP.0000000000000558 30557276

[B30] Wong A. Bhyat R. Srivastava S. Boissé Lomax L. Appireddy R. (2021). Patient care during the COVID-19 pandemic: use of virtual care. J. Med. Internet Res. 23:e20621. 10.2196/20621 33326410 PMC7822645

